# Attitudes towards using single-pill combination (polypill) therapy in heart failure: patients' and physicians' perspectives

**DOI:** 10.1093/eschf/xvag044

**Published:** 2026-02-11

**Authors:** Jan Biegus, Rafał Tymków, Javed Butler, Marco Metra, Ovidiu Chioncel, Vijay Chopra, Marianna Adamo, Julio Nuñez, Giuseppe Rosano, Clara Saldarriaga, Michael Böhm, Shelley Zieroth, Piotr Ponikowski

**Affiliations:** Jan Mikulicz Radecki University Hospital in Wrocław, Department of Cardiology, Borowska 213, Wroclaw 50-556, Poland; Department of Cardiology, Clinical Department of Intensive Cardiac Care, Faculty of Medicine, Institute of Heart Diseases, Wroclaw Medical University, Borowska 213, Wroclaw 50-556, Poland; Jan Mikulicz Radecki University Hospital in Wrocław, Department of Cardiology, Borowska 213, Wroclaw 50-556, Poland; Baylor Scott and White Research Institute, Dallas, TX, USA; University of Mississippi, Jackson, MS, USA; Institute of Cardiology, ASST Spedali Civili, Department of Medical and Surgical Specialties, Radiologic Sciences and Public Health, University of Brescia, Brescia, Italy; Emergency Institute for Cardiovascular Diseases “Prof. C.C.Iliescu”, University of Medicine “Carol Davila,” Bucharest, Romania; Department of Cardiology, Max Super Speciality Hospital, Saket, New Delhi, India; Institute of Cardiology, ASST Spedali Civili, Department of Medical and Surgical Specialties, Radiologic Sciences and Public Health, University of Brescia, Brescia, Italy; Department of Cardiology, Hospital Clínico Universitario de Valencia, Valencia, Spain; Department of Medicine, Valencia, Spain; Department of Human Sciences and Promotion of Quality of Life, San Raffaele Open University of Rome, Rome, Italy; Department of Clinical Cardiology, IRCCS San Raffaele Roma, Rome, Italy; Department of Cardiology CardioVID CLINIC, Pontificia Bolivariana University, Medellín, Colombia; Medical Faculty, HOMICAREM (HOMburg Institute for CArdioREnalMetabolic Medicine) and Klinik für Innere Medizin III, Saarland University, Homburg/Saar 66421, Germany; Section of Cardiology, Max Rady College of Medicine, University of Manitoba, Winnipeg, MB, Canada; Jan Mikulicz Radecki University Hospital in Wrocław, Department of Cardiology, Borowska 213, Wroclaw 50-556, Poland; Department of Cardiology, Clinical Department of Intensive Cardiac Care, Faculty of Medicine, Institute of Heart Diseases, Wroclaw Medical University, Borowska 213, Wroclaw 50-556, Poland

**Keywords:** GDMT, Polypill, Single-pill combination

## Abstract

**Introduction:**

Single-pill combinations (SPC, *polypills*) have proven effective in cardiovascular areas, yet no such therapy exists for patients with heart failure (HF) despite substantial polypharmacy and pill burden in this population. Simplifying treatment through an HF-specific SPC containing key guideline-directed medical therapy (GDMT) components could improve adherence and outcomes.

**Methods:**

Two prospective, electronic surveys were conducted between June and October 2025 to assess real-world attitudes towards a polypill in HF with ejection fraction ≤50%. The physician-oriented survey (22 questions) was distributed internationally and explored GDMT practices, perceived needs, barriers, and potential preferred composition of an HF dedicated SPC. The patient-oriented survey (11 questions) explored medication burden, adherence, and perceptions of a potential polypill use.

**Results:**

A total of 250 physicians and 126 patients participated. Among physicians, 77% reported a clear need for strategies to simplify GDMT optimisation in HFrEF, with cost (66%) and polypharmacy (54%) being selected as the most frequent barriers. Nearly all physicians (95%) recognized a real clinical need for an HF-specific SPC, and most perceived it as clinically useful (88%), logistically feasible (76%), and acceptable to patients (94%). Approximately 48% of physicians declared that they would use it regularly, and another 49% would use it in selected patients. The preferred composition of HF-specific SPC included a beta-blocker, mineralocorticoid receptor antagonist (MRA), and SGLT2 inhibitor (61.2%).

Among patients, polypharmacy was common (70% taking ≥6 drugs daily), and 75% admitted to occasional non-adherence. Most responders (82%) would support a solution that reduces the pill burden, and 83% would take an HF-specific SCP if offered, particularly if there is no extra cost.

**Conclusion:**

Both physicians and patients showed strong openness and willingness towards an HF-specific SPC, supporting further development and evaluation of HF-specific polypill strategies.

## Graphical Abstract

**Graphical Abstract:**
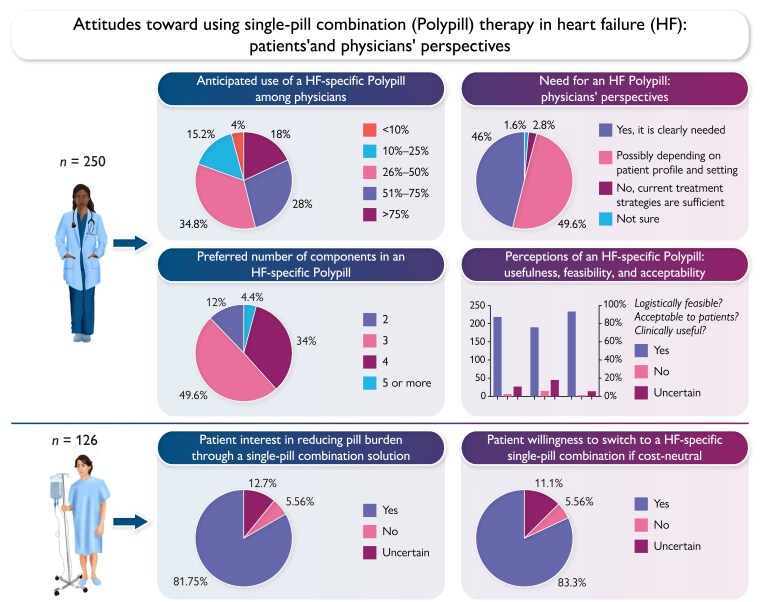
Attitudes toward using single-pill combination therapy in heart failure: patients' and physicians' perspectives.

## Introduction

Single-pill combination (SPC, polypill) therapy has been shown to be useful and effective in several cardiovascular areas, such as hypertension, coronary atherosclerotic disease, and prevention.^1–4^ However, polypills for heart failure (HF) patients are not. This contrasts with the fact that HF seems to be a perfect setting for a polypill approach, as polypharmacy is common and the pill burden is substantial.^5^ HF patients, particularly those with HFrEF (HF with reduced ejection fraction), are recommended to take drugs from at least four (if not five) major drug classes.^6^ Therefore, the polypill concept appears highly attractive for these patients. From a theoretical perspective, the polypill may provide several benefits over the standard approach, with each drug being prescribed as a separate pill, from both the patient’s and the physician’s perspective. These include, but are not limited to, greater ease of administration and a reduced pill burden, resulting in improved treatment adherence, which, in the long term, may translate to improved outcomes.

This study aimed to examine the real-world attitudes of patients and physicians toward the single-pill combination concept in HF.

## Methods

We conducted two separate prospective, electronic surveys to examine attitudes toward the polypill concept. The physician-oriented survey was written in English and contained five major domains: respondent information, HF guideline-directed medical therapy (GDMT) treatment questions, polypill questions, polypill in HF—potentially optimal composition, and the potential population for the polypill questions. The physician's survey consisted of 22 questions in total. In the introduction to the survey, respondents were informed that the survey pertained to the management of patients with HF and a reduced or mildly reduced ejection fraction (LVEF <50%). The survey is available at: https://forms.gle/Fi8wfKUR5RuifhTTA. The authors of the paper disseminated the physician survey within their professional community. The survey was disseminated from June 2025 to October 2025.

The patient-oriented survey was written in Polish, and only Polish patients responded. It included three main areas: questions to characterize the respondent, the respondent's pill burden and adherence, and their opinion on the polypill concept, with a total of 11 questions. The patient’s survey was deliberately short to make it more responder-friendly. We gathered the survey responses from June 2025 to October 2025. The survey is available at: https://forms.gle/r9K6RCShnGSWWRig7. All participants’ responses were kept anonymous, and their participation was voluntary.

The data are presented as numbers and percentages of the total population. No statistical analyses were performed.

## Results

### Physician-targeted survey

From June 2025 to October 2025, a total of 250 physicians responded to the survey. The majority of them were from Europe (*n* = 207, 82.8%) and South/Central America (8%); the other regions included North America and Asia. The majority of responders were cardiologists (80.4%), HF specialists (8.4%), and internists (5.2%). The responders had significant clinical experience (>20 years of practice declared by 24%, 11–20 years of practice: 30.4%, 5–10 years of practice: 22.8%, and <5 years: 22.8%). The physicians worked primarily in academic hospitals (77.6%) or public/non-academic facilities (13.6%). The majority of responders managed a substantial number of HFrEF/HFmrEF patients per week: 31–50/week (8.8%), 21–30/week (14.4%), 10–20/week (46.4%), <10/week (25.2%).

A majority of physicians (*n* = 193, 77.2%) agreed ‘definitely’ with the statement that ‘there is a need for strategies to improve and simplify the optimisation of GDMT in patients with HFrEF/HFmrEF’, while the rest answered ‘possibly’. The most common barriers to the optimal implementation of GDMT in HF reported by the responders were cost of medications (*n* = 164, 65.5%), polypharmacy (*n* = 134, 53.6%), adverse effects or drug intolerance (*n* = 118, 47.2%), and insufficient follow-up opportunities for medication optimisation (*n* = 110, 44%).

**Figure 1:**
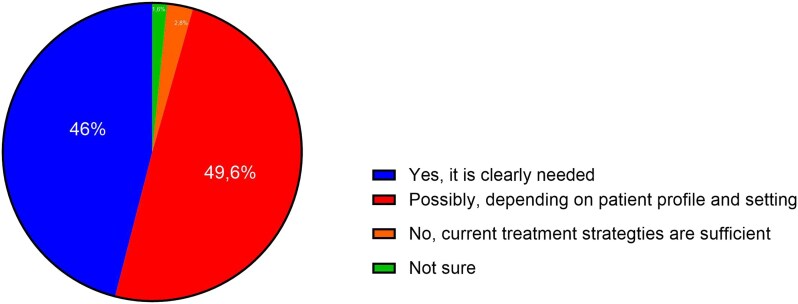
Perceived need for an HF-specific single pill combination among physicians. Responses to the question: ‘Do you think there is a need for a polypill combining key GDMT components for patients with heart failure with reduced/mildly reduced ejection fraction (LVEF <50%)?’ (*n* = 250)

**Figure 2:**
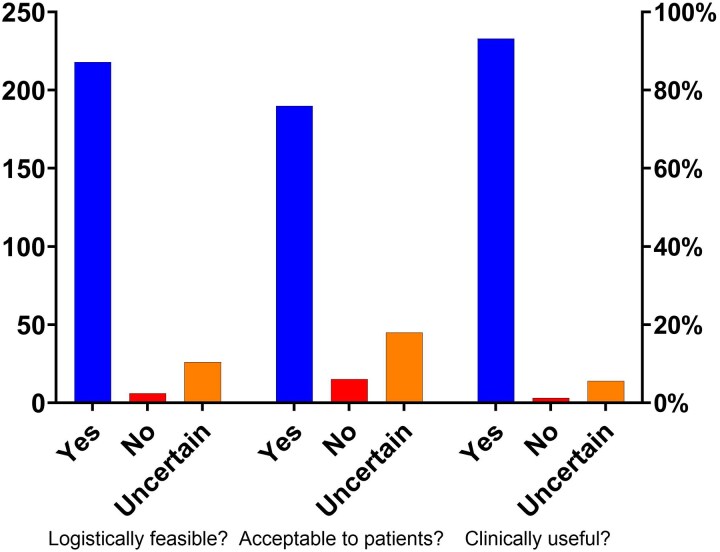
The anticipated clinical usefulness, feasibility, and patient acceptability of an HF-specific single-pill combination

The majority of respondents (49.6%) recognized a clear need for an SPC that combines key GDMT components for patients with HF and LVEF <50%. The other 115 physicians (46%) indicated that the need might depend on the patient's profile (*Figure 1*). Almost all responders (*n* = 226, 90.4%) agreed (49.2%) or strongly agreed (41.2%) that the HF-specific SPC would improve adherence. Most respondents perceived the polypill as clinically useful (*n* = 218, 87.2%), logistically feasible (*n* = 190, 76.0%), and acceptable to patients (*n* = 233, 93.2%) (*Figure 2*).

A total of 123 physicians (48.4%) indicated they would use the HF polypill regularly, 121 (49.2%) would use it only in selected patients, while just 3 (1.2%) responded negatively, and 3 (1.2%) were unsure. The top three pieces of evidence that responders considered essential to support the use of the polypill in their practice were: real-world data confirming higher adherence and safety (*n* = 155, 62%), a randomized controlled trial demonstrating clinical efficacy (*n* = 137, 54.8%), and health-economic data showing cost-effectiveness (*n* = 95, 38%).

**Figure 3:**
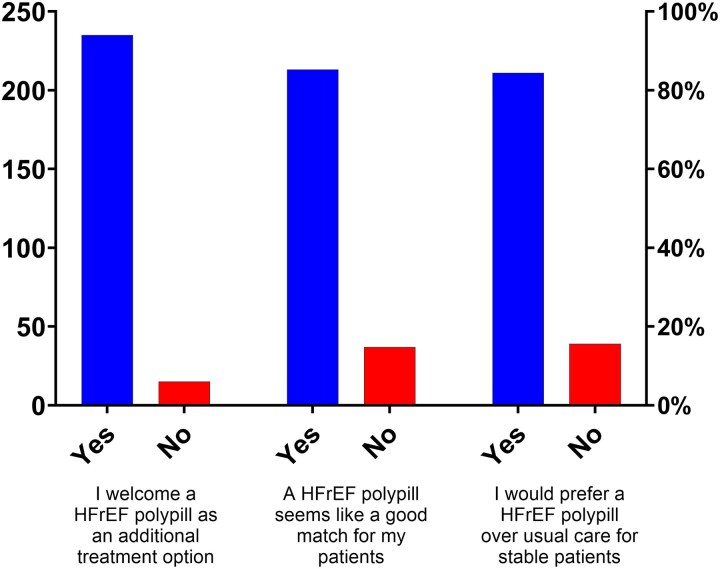
Physician support for the concept of an HF-specific SPC

The top three concerns regarding the use of the polypill in HF were: tolerability/safety concerns (*n* = 161, 64.4%), reimbursement and cost (37.6%), and limited clinical trial evidence (18.8%). Most of the respondents support the concept of a polypill in HF patients, with 235 (94%) welcoming a polypill as an additional treatment option, *n* = 213 (85.2%) felt that polypill was a good match for their patients, and *n* = 211 (84.4%) would prefer HF-specific SPC over the usual care for stable patients (*Figure 3*).

**Figure 4:**
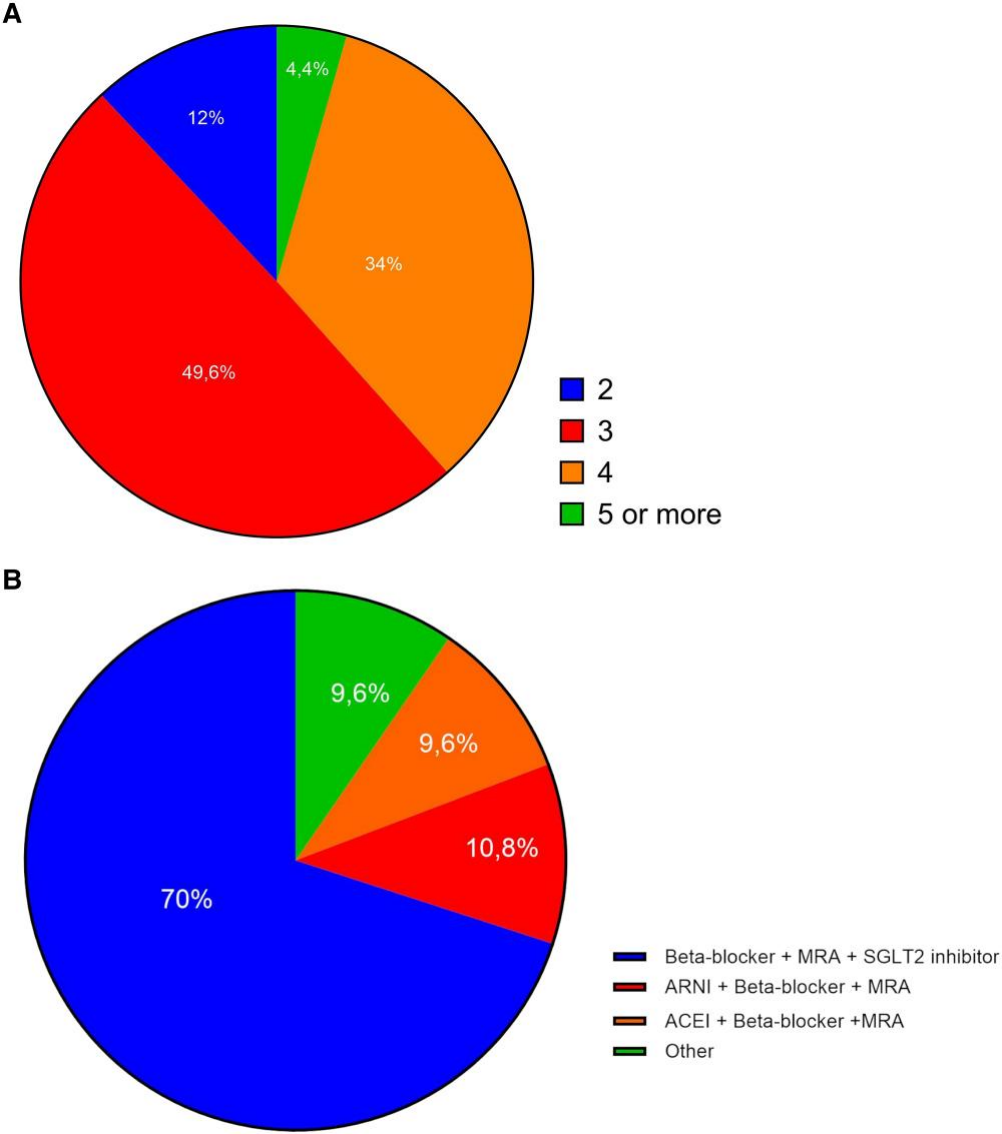
The components of the single-pill combination. (*A*) The proposed number of active components the potential HF-specific SPC should contain. (*B*) The proposed ‘optimal’ components of the HF-specific SPC

Most physicians indicated that the polypill should contain three components (49.6%), while 85 (34%) responders selected four components, 30 (12%) selected two components, and the remaining 11 (4.4%) selected five components (*Figure 4A*). The top selected combination of the GDMT that HFrEF/HFmrEF polypill should contain was beta-blocker + MRA + SGLT-2i (*n* = 153, 61.2%), ARNI + MRA + SGLT-2i (*n* = 70, 28%), and beta-blocker + ACEI + SGLT-2i (*n* = 64, 25.6%). The same combination (beta-blocker + MRA + SGLT-2i) was indicated as the most likely considered as a treatment option for patients with HFrEF by 175 (70%) of the responders (*Figure 4B*).

Regarding the timing of the potential prescribing of the polypill ‘the outpatient/community setting, once a patient is on stable doses of GDMT’ was the most frequently selected option, followed by the ‘at time of hospital discharge’ and ‘during hospitalization’, *n* = 188 (75.2%); *n* = 109 (43.6%) and *n* = 71 (28.4%), respectively. The polypill would be most strongly considered by physicians for patients with poor adherence (*n* = 174, 69.9%), patients with polypharmacy (*n* = 161, 64.4%), and in all HF patients (*n* = 91, 36.4%). On the other hand, the top three selected populations for whom the polypill would not be suitable or indicated were: frail patients (*n* = 115, 46%), patients with multiple comorbidities (*n* = 108, 43.2%), and the elderly population (*n* = 71, 28.4%).

**Figure 5:**
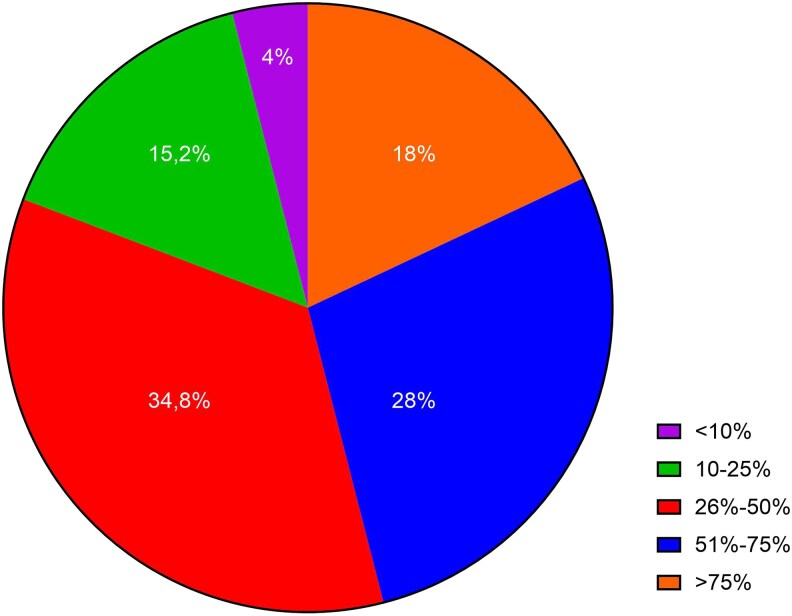
The anticipated use of the polypill. Estimated proportion of HFrEF patients who could be offered an HF-specific SPC. Responses to the question: ‘*To what percentage of your HFrEF patients would you offer the polypill?’*

The largest proportion of respondents (34.8%) estimated they would offer the polypill to 26–50% of their HFrEF patients, followed by (28.0%) for 51–75%, (18.0%) for >75%, and (15.2%) for 10–25%, while only a minority (4%) selected <10% (*Figure 5*).

### Patient-targeted survey

A total of 126 patients responded to the survey. The polypharmacy was very frequent. The majority of patients (*n* = 88, 69.8%) reported receiving ≥6 medications daily, 21 (16.7%) selected five medications, and the remaining 17 (13.3%) declared taking ≤4 medications daily. The medications were taken most often twice daily (*n* = 59, 46.8%), followed by three times per day (*n* = 55, 43.7%).

Almost ¾ of patients reported missing their medications, with the frequency categorized as rarely (defined as 1–2 times per month) (46.8%); sometimes (defined as 1–2 times per week) (18.3%); or frequently (>3 times per week) (4.8%). The remaining 38 (30.2%) patients declared that they never miss their medications. Most responders (58.7%) indicated that daily medication intake was not an issue; however, 31% found it problematic, and 10.3% were unsure and chose ‘hard to say.’ Analogously, the frequency of medication intake was not perceived as a significant burden by 64 patients (50.8%). In contrast, 40.5% selected it as a significant burden, and 8.7% chose the ‘hard to say’ answer. Notably, 25 patients (19.8%) reported missing some of their medications due to the number or high frequency of intake.

Forty-five (35.7%) patients selected that the high number of medications taken daily negatively impacts their well-being or everyday living activities, while *n* = 15 (11.9%) had no opinion on this issue.

**Figure 6:**
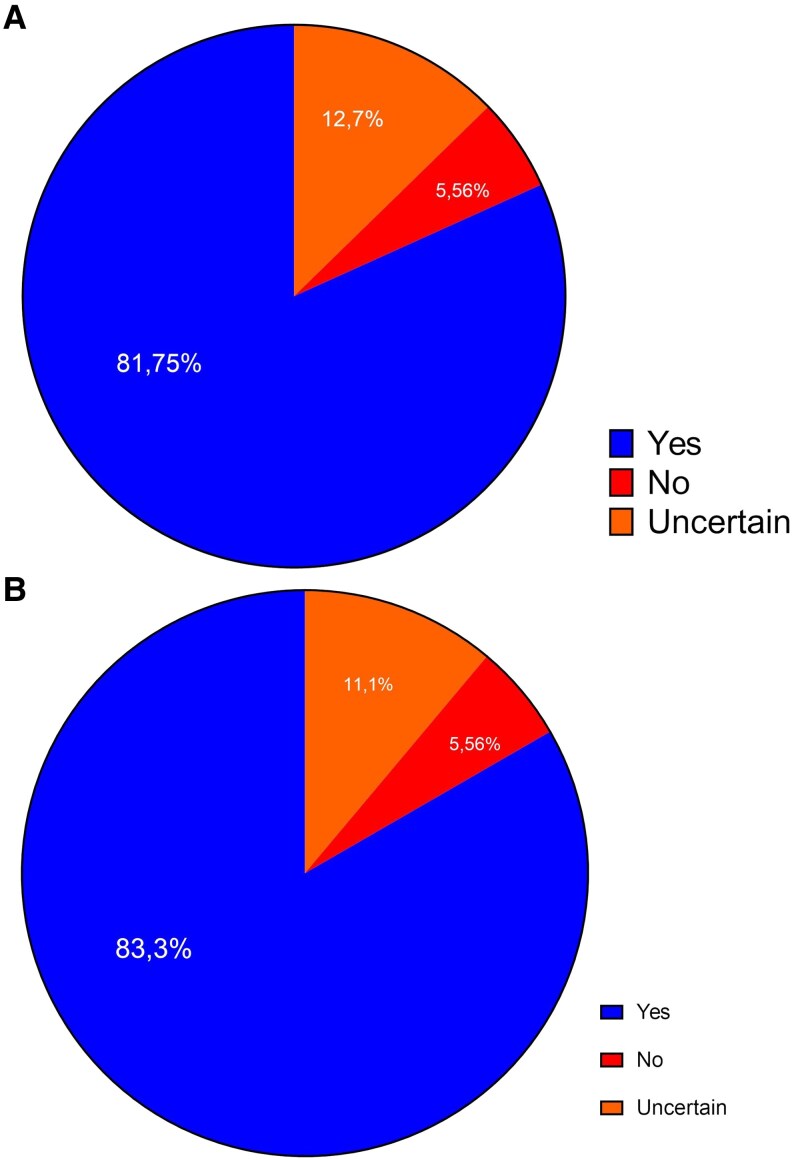
Patient’s perspective on single-pill combination. (*A*) Patient interest in reducing pill burden through a single-pill solution. Responses to the question: ‘*Would you be interested in a solution that allows you to take fewer pills per day (e.g. one tablet containing several medications)?’* (*B*) Patient willingness to switch to an HF-specific SPC if cost-neutral. Responses to the question: ‘*If your doctor offered to replace several of your current medications with a single pill containing the same substances (a so-called polypill), would you agree if it did not involve any additional cost?’*

Importantly, the majority of patients (81.7%) expressed interest in solutions that reduce the number of tables daily (i.e. SPC) (*Figure 6A*). Moreover, 87 responders (69%) would like their physician to offer them a solution that simplifies adherence and compliance, while only five responders (4%) would not welcome it. Most patients (*n* = 105, 83.3%) would like their physician to offer them a polypill, and they would be likely to take it if there were no additional cost associated with the solution. While only 5.6% would not like that offer, and 11.1% selected ‘hard to say’ answer (*Figure 6B*). Lastly, the majority of patients (57.1%) believe that the polypill would improve their everyday medication adherence, while 22.2% think it would not change adherence, and 20.6% have no opinion on the matter.

## Discussion

The two surveys demonstrated that both physicians and patients favour polypills for therapeutic simplification. Notably, the majority of physicians (∼77%) reported a need for strategies to improve and simplify the optimisation of GDMT in patients with HFrEF/HFmrEF. This indeed seems to be an unmet need in the pharmacotherapy of HF patients. At the same time, we must emphasize that polypharmacy (the need to take ≥5 medications) was the second most common (after cost of medications) barrier that the physicians selected to optimize the implementation of GDMT. The patients` reluctance to take a high daily pill burden was selected by 37% of responders. When we appreciate that HF patients often have several comorbidities, and sometimes also take other medications and supplements without clear medical indications, then we realize that the pill burden in HF is substantial.^7,8^

All the above most likely resulted in a strong openness to the single-pill combination concept, with 95% of physicians recognising the real clinical need for a polypill combining key GDMT components for HF patients with reduced or mildly reduced ejection fraction (LVEF <50%). Notably, about 46% of respondents clearly recognized the need, whereas ∼49% noted that it depends on the patient’s profile and clinical context. This is also supported by the fact that most physicians believe the SPC would improve adherence, be clinically useful, logistically feasible, and acceptable to patients. Given that, almost all responders declared that they would use the HF-specific SPC in their everyday practice, either regularly (∼49%) or only in selected patients (∼48%).

Despite the evident anticipation that the polypill concept is needed for HF patients, the respondents pointed out that the main concerns regarding its use were tolerability and safety issues, cost, and the lack of clinical evidence for its effectiveness. These comments highlight the relative lack of familiarity of the physicians with the regulatory pathways for SPCs, as these combinations can be registered as both substitution therapy and as an initial approach. In the first case, no additional data is required because patients are switched from two or more drugs at a given dose to an SPC containing the same doses of the same medications. The effectiveness of an SPC containing medications currently used for the given disease is not needed if the medicines contained in the SPC are used in common practice and/or suggested by guidelines.

Moreover, the physicians would be less likely to initiate the SPCs in frail patients, in patients with multiple comorbidities, and in elderly patients.^9^ On the other hand, almost half of the responders selected that they would use the polypill in more than half of their patients.

Moreover, although we fully agree that the above-mentioned vulnerable populations certainly require special caution during treatment, it is worth noting that in the STRONG-HF trial—where patients received high-intensity care (although, of course, this is not equivalent to a polypill approach)—no significant increase in risk was observed among elderly patients or those with multiple comorbidities.^10–12^

Interestingly, the majority of physicians selected that the polypill should contain three (not four) components, and those suggested components were: beta-blocker, MRA, and SGLT-2i. This is probably a very pragmatic option, as the inclusion of ARNi (or some ACEI), which should be taken twice daily, would be problematic in terms of simplifying the daily regimen. On the other hand, including a loop diuretic, whose dose might need adjustment based on the patient's fluid status, would make the single-pill combination less pragmatic.

The patient survey confirmed that HF patients’ pill burden is significant, which may contribute to reduced adherence. Interestingly, only about one-third of patients reported that they never forget to take their medications, while the other report some non-adherence. However, it should be noted that for more than half of the respondents, the number of medications taken daily was not perceived as a significant issue. At the same time, one-third of patients indicated that the high number of medications negatively affects their everyday life and overall well-being.

Surprisingly, patients expressed significant openness to the novel concept of the polypill. The vast majority of patients expressed interest in a solution that would allow them to take fewer pills daily, and as many as 83% would like their physician to offer them a polypill for HF treatment. Lastly, nearly 60% of patients believed that such a solution would improve their daily medication adherence.

Lastly, our study is the first to capture the real-world attitudes and opinions of the two most important stakeholders involved in prescribing and taking medications. We are not aware of any previous studies that have examined these perspectives simultaneously. The views and behaviours of both groups are crucial for the potential success of the HF-specific SPC concept, which, from a scientific standpoint, represents an unmet need for HF patients.

In conclusion, physicians and patients demonstrated strong openness and anticipation toward an HF-specific SCP concept. The perceived benefits of adherence and treatment simplification might outweigh concerns regarding safety and cost. Both physicians and patients clearly declared their willingness to use the HF-specific SPC if it were available.

### Limitations

Most respondents to the survey were physicians from Europe; therefore, caution is necessary when applying the results to other populations because prescribing patterns and medication availability vary across regions.^13–15^ Additionally, there is always some selection bias in all volunteer surveys, which also affects our research. The patient-focused examination only studied the Polish population, which presents another limitation. Our study is limited to the information that was predefined by the survey questions and, in most cases, suggested in the survey answers. The survey did not ask specific questions about the number of doses within pre-defined combinations. The survey neither asked questions that could distinguish the patient’s characteristics associated with higher versus lower willingness to take polypills.

## Declarations

### Data Availability

The data that support the findings of this study are available from the corresponding author upon reasonable request.
